# Physical activity interventions for hospitalised people living with dementia: systematic review and meta-analysis

**DOI:** 10.1007/s41999-025-01304-x

**Published:** 2025-09-16

**Authors:** Emma Elliott, Jodi Ventre, Sarah Kate Smith, William Carey, Charlotte Eost-Telling, Annemarie Money, Toby Bryce-Jones, Victoria Dickens, Chris J. Todd, Emma R. L. C. Vardy

**Affiliations:** 1 NIHR-ARC Greater Manchester, School of Health Sciences, The University of Manchester, The University of Manchester, Jean McFarlane Building, Oxford Road, Manchester, M13 9PL UK; 2 Manchester Academic Health Science Centre, Manchester, M13 9NQ UK; 3 Northern Care Alliance NHS Foundation Trust, Salford, UK; 4 University Hospitals of Leicester NHS Trust, Leicester, UK; 5 Manchester University NHS Foundation Trust, Manchester, M13 9WL UK

**Keywords:** Dementia, Hospitalisation, Deconditioning, Physical activity, Exercise, Functional decline

## Abstract

**Purpose:**

People living with dementia are vulnerable to adverse outcomes of hospitalisation, such as functional decline and deconditioning. The aim of this review was to examine the effectiveness of hospital-based interventions involving physical activity on deconditioning outcomes in people living with dementia.

**Methods:**

Five databases (MEDLINE, CENTRAL, Embase, PsycINFO, and CINAHL) were searched to January 2025 to identify eligible studies. Two researchers independently carried out screening, data extraction and quality assessment. Inclusion criteria: adults with a diagnosis of dementia, acute hospital admission, and interventions involving physical activity during hospitalisation. Data were pooled for strength, balance, functional outcomes, readmission, falls, length of stay, and delirium. We used GRADE to assess the certainty of evidence.

**Results:**

2179 records were identified and six studies (*n* = 576 with dementia) included: one randomised-controlled trial, five non-randomised or quasi-randomised. Studies were conducted in an “acute care for elders unit” (*n* = 2), acute psychogeriatric ward (*n* = 2), general medical unit (*n* = 1), and geriatric rehabilitation ward (*n* = 1) across six countries. In meta-analyses, there was less decline in basic activities of daily living at 3 months in the physical activity group (change from pre-admission status); two studies, mean difference 1.27 (95% CI 0.36–2.18) but there were no differences found in all other outcomes at discharge or 3 months, compared to usual care. Certainty of the evidence is low/very low.

**Conclusion:**

Due to the limited available evidence, there is uncertainty about the effect of physical activity interventions above usual care. More high-quality research is needed to improve outcomes for people living with dementia in hospital.

**Supplementary Information:**

The online version contains supplementary material available at 10.1007/s41999-025-01304-x.

## Key summary points

**Aim:**

The aim of this review was to examine the effectiveness of hospital-based interventions involving physical activity on deconditioning outcomes in people living with dementia.

**Findings:**

In pooled analyses, there was less decline in independence in basic activities of daily living at 3 months (change from pre-admission status) in the physical activity group, but there were no differences in all other outcomes at discharge or 3 months, compared to usual care. Certainty of this evidence is low or very low, so results might be expected to change if additional high-quality studies are conducted.

**Message:**

There is uncertainty about the effect of physical activity interventions above usual care and more high-quality work with people living with dementia is needed.

**Supplementary Information:**

The online version contains supplementary material available at 10.1007/s41999-025-01304-x.

## Introduction

People living with dementia are more frequently admitted to hospital than those without, independent of physical comorbidities [1]. They have longer lengths of stay [2], and higher rates of mortality, admissions to care home [3], and delirium [4].

Deconditioning refers to decline in physical, cognitive, and functional abilities, as a result of prolonged inactivity, such as bed rest [5, 6]. Deconditioning is a particular concern for hospitalised patients [7]; low in-hospital mobility is associated with functional deterioration at discharge and follow-up [8, 9], with people with cognitive impairment being at a heightened risk [10, 11]. People with cognitive impairment have very low levels of physical activity in hospital [12], for example spending an average of 84% of their time being sedentary [13].

Preventing hospital-associated deconditioning is the aim of campaigns, such as ‘End PJ paralysis’, ‘Get Up, Get Dressed, Get Moving’, and ‘Recondition the nation’, all emphasising the importance of movement in hospital. Previous reviews have examined the effectiveness of physical activity interventions in the hospital setting, but the target population has not had a dementia focus. An umbrella review [14] addressing interventions to promote physical activity and reduce functional decline in hospital included 12 reviews. The evidence suggests that progressive mobilisation interventions and multicomponent interventions are effective in improving participation and reducing functional decline in hospital. The evidence for exercise alone, however, has been inconclusive [15]. A review examining rehabilitation in older adults who had already deconditioned due to acute hospitalisation [16] found positive results from individual studies for activities of daily living (ADLs) and discharge destination, but pooled analyses could not be performed. Existing reviews have not included subgroup analyses for people living with dementia, so it is not clear if they have been included in the previous studies, if they adhere to interventions or what evidence exists regarding effectiveness in this population.

Systematic reviews have also found evidence that exercise can be beneficial for people with dementia outside the hospital setting, for example physical functioning [17–19]. Benefits in physical function have also been seen in the residential care setting [20]. However, the hospital setting brings specific challenges to implementing such an intervention. These include challenges specific to dementia, such as greater levels of supervision needed, staff being risk averse (due to risk of falls), and views regarding rehabilitation potential [21], and also barriers due to the hospital environment (chaotic, crowded, lack of freedom) and staffing/resources [22, 23].

The aim of this review was to examine the effectiveness of interventions involving physical activity delivered in the hospital setting for preventing deconditioning, in people living with dementia.

## Methods

This systematic review was conducted adhering to the Preferred Reporting Items for Systematic Reviews and Meta-Analyses (PRISMA) guidelines, and the protocol was prospectively registered on Prospero: CRD42023482947.

### Search strategy and selection criteria

Five databases (MEDLINE, the Cochrane central register of controlled trials [CENTRAL], Embase, PsycINFO, and CINAHL) were searched from inception to 8th December 2023 and updated until 22nd January 2025 to identify eligible studies. Randomised and non-randomised-controlled trials were included. Inclusion criteria were adults with a documented diagnosis of dementia (all aetiologies), admitted to hospital following an acute event. Interventions involving physical activity (including but not restricted to exercise) during the hospitalisation period were eligible. Full search strategy is available in Supplementary methods 1 and full inclusion criteria in Supplementary methods 2.

Two review authors independently carried out screening (EE and JV or SS) and data extraction (EE and WC), with disagreements resolved by a third review author (CET). All screening was conducted in Rayyan (https://www.rayyan.ai/) and the reference lists of included studies and relevant published systematic reviews were screened. Where studies included participants where only a subgroup had dementia, study authors were contacted to request unpublished data.

We extracted data on hospital setting, country, demographic information, intervention and control details, and outcome data.

### Primary outcomes

Eligible studies needed at least one of the following outcome measures which we considered to be direct or proxy measures of deconditioning: strength (strength and muscle mass), aerobic or exercise capacity, and functional ability (mobility, balance, basic, and instrumental ADLs). We included measures that were performance-based, clinician-reported, or caregiver/patient-reported.

A minimum of two timepoints of assessment were required for inclusion: baseline assessment had to be pre-admission status or at admission/start of intervention and a follow-up assessment either during admission (e.g., at discharge) or within 3 months post-discharge.

### Secondary outcomes

We also extracted data on the following process and outcome measures: adverse events (e.g., falls, pressure ulcers, and delirium), frailty, length of stay, discharge destination, mortality, readmission, quality of life, and mood.

### Quality assessment and certainty of the evidence

Two review authors (EE and JV or WC) independently assessed risk of bias using the Cochrane ROB-2 tool [24] and the certainty of the evidence for each outcome using GRADE [25] in GRADEpro [26].

### Meta-analysis

Random-effects meta-analyses were run where outcome data were available for ≥ 2 studies. Data were combined using mean difference if the same measure was used and standardised mean difference where they differed (including modified versions of the same measure). Change from baseline data were used for continuous outcomes, due to most studies not using randomised allocation (therefore, there may be baseline differences in participants) and baseline status is important to examine evidence of deconditioning. Where studies had two baseline values (pre-admission and admission/before intervention), separate analyses were run using both. Participants were excluded from the sit-to-stand analysis (measured in seconds) or gait speed analysis (measured in cm/seconds), where a score of 0 (unable to complete) was recorded in the provided dataset at either timepoint.

Where standard deviations of change from baseline scores were not available, they were imputed using another study where full data were available to calculate the correlation coefficient. Data from randomised and non-randomised (including quasi-randomised) studies were not pooled. Dichotomous outcomes were falls (yes/no), readmission to hospital (yes/no), and inpatient delirium (yes/no). Analysis was performed in RevMan (online version). Forest plots were used to present data, and statistical heterogeneity was assessed using the I^2^ statistic.

## Results

**Fig. 1:**
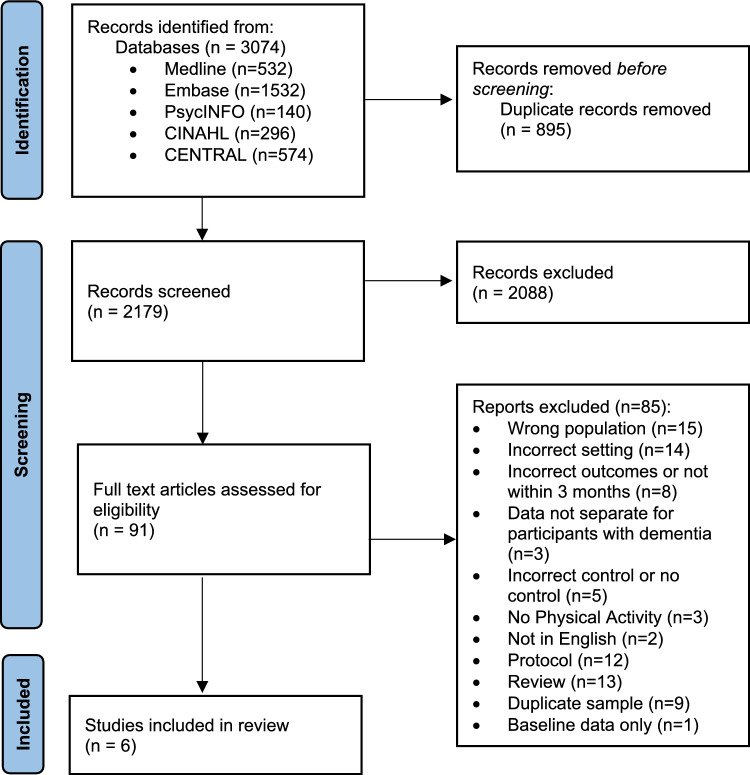
PRISMA flowchart

2179 records were identified, and six studies (576 participants with dementia) were included (Fig. 1 for PRISMA flowchart and Supplementary Table 1 for PRISMA checklist).

One was a randomised-controlled trial [27], and five were non-randomised or quasi-randomised-controlled trials [28–32]. The trials were conducted in the following settings: “acute care for elders” (ACE) unit (*n* = 2), acute psychogeriatric ward (*n* = 2), general medical unit (*n* = 1), and geriatric rehabilitation ward (*n* = 1). Dementia severity was largely mild-moderate (severe recorded in 1 study [33]). Aetiology of dementia was reported in two studies: more than half of the sample with Alzheimer’s disease (AD) (59% and 71%) [30, 33]. Comorbidities were reported in five studies: four reported the Charlson comorbidity index (three studies reported mean scores: ranged 3–7, one study just reported the range: 1–5), one reported the Comorbidity Illness Rating Scale (mean score 12). The six studies were conducted in six different countries: Spain, Switzerland & Belgium, Germany, Finland, and Australia. Three study authors [29, 31, 32] provided individual patient level data of participants with dementia for inclusion in the review.

See Table 1 for details of the included studies. Reasons for exclusion of selected excluded studies are presented in Supplementary Table 2.

**Table 1:** Included studies

Study	Ward	Number of participants with dementia	Age (mean)	Sex (% female)	Dementia aetiology	Dementia severity
*Randomised*						
Burge 2016	Acute psychogeriatric ward	160	IG 81.7 CG 81.1	IG 49% CG 54%	59% AD 18% Mixed 4% Vascular 3% Frontotemporal 3% Lewy body 1% Subcortical	Moderate–severe CDR 3: IG 32%, CG 34%, MMSE score ≤ 20: 84% IG, 78% CG
*Non-randomised or quasi-randomised*						
Mudge 2008*	General medical unit	11	IG 82.2 CG 87.8	IG 33% CG 60%	NR	NR
Ortiz-Alonso 2020	Acute care for elders unit	63	IG 87.8 CG 87.4	IG 67% CG 47%	NR	Severe dementia excluded
Pitkanen 2019*	Acute psychogeriatric ward	175	IG 78.0 CG 77.7	IG 48% CG 62%	71% AD 11% Vascular 18% Other or Not specified	NR, MMSE IG 13.2 (7.2), CG 13.4 (7.9)
Rodriguez-Lopez 2024*	Acute care for elders unit	37	IG 88.1 CG 86.8	IG 47% CG 75%	NR	Severe dementia excluded
Schwenk 2014	Rehabilitation ward at geriatric hospital	130	IG 84.2 CG 83.9	IG 84% CG 76%	NR	Mild to moderate stated. Low MMSE excluded. MMSE IG: 21.4 (2.6), CG: 22.2 (2.3)

AD: Alzheimer's disease, CDR: Clinical Dementia Rating, CG: Control Group, IG: Intervention Group, MMSE: Mini-Mental State Examination, NR: Not recorded

*Multicomponent interventions

### Interventions

All physical activity interventions were classed as exercise (a form of physical activity that is planned, structured, and repetitive) [34]. Three studies included a single component intervention [28, 32, 33], and three studies included exercise as part of a multicomponent intervention [29–31] (other components were music-based: *n* = 1, cognitive stimulation and education of ward staff: *n* = 1, and health education sessions and monthly telephone counselling post-discharge: *n* = 1). Different types of exercise were included; all studies included walking and a strength/resistance component (e.g., strength training, sit-to-stand exercises). Other elements included balance (4 studies) and flexibility (2 studies). Two interventions were group-based, three were individual, and one study included both. Interventions were supervised by staff (e.g., physiotherapist) in all studies.

Frequency of sessions offered was a minimum of once daily on weekdays, with most studies offering > 1 session/day. Session duration ranged 20–45 min across studies or were tailored to the individual. Intensity of the interventions were described in two studies: in Schwenk et al., resistance training was described at sub-maximal intensity (70–80% of the one-repetition maximum) [28] and in Burge et al., they aimed to achieve moderate intensity [27]. The interventions typically started soon after admission (except for Burge et al., which started 2 weeks after). Intervention duration was 4 weeks in one study [27], and based on length of stay in other studies, with one study aiming for the intervention to continue at home unsupervised following discharge [31]. The amount of physical exercise received by participants with dementia was available in four studies. Two studies reported mean trained days: 2.6 days [32] and 12.5 days [28]. Two studies reported mean number of sessions received: 7 [30] and 13 [33]. In terms of adherence, across three studies, the number of trained days divided by the days available for training was reported: 56% [32], 66% [33], and 69% [28]. One study recorded that 5/15 (33%) of the intervention group had > 50% adherence in hospital and > 50% at home following discharge [31]. One study reported that the type of dementia was significantly associated with adherence; those with Alzheimer’s disease were more adherent than those with other types of dementia [33].

All intervention groups received usual care in addition to the intervention.

### Control group

In all studies, usual care was included in the control group, which could include rehabilitation and/or mobilisation. On the rehabilitation ward participants received occupational therapy, speech therapy and physiotherapy [28]. On the general medical unit, usual care from the multidisciplinary care team included daily discussion of progress and discharge plan and referral to the physiotherapist or occupational therapist if there were concerns about mobility or function [29]. Usual care on the ACE unit was described in one study as getting patients out of bed every day, avoiding fluid therapy, removing catheters where possible, initiating mobilisation in the room and ward corridors and reminders to do so every day, changing the position in bed every 3 h if mobilisation is not possible, avoiding physical restraints [31]. On one acute psychogeriatric ward, usual care included some recreational activities (e.g., reading newspaper, games, painting, listening to music, and exercise training). It is noted that these activities were carried out infrequently based on nurse’s interests and availability. On some days, it was also possible to walk outdoors in a group [30]. On the other acute psychogeriatric ward, usual care included sensory interventions, structuring of the day, and medical/systemic interventions. In addition, a social activity programme (watching videos or playing together) was offered to the control group [33].

Two studies recorded details on sessions of usual care rehabilitation received. One study reported a similar number of physiotherapy sessions in both the control and intervention arms [29], whereas, in another study, the control group received a longer duration of usual care rehabilitation than the intervention group, due to time overlap with the intervention [28].

### Risk of bias

The risk-of-bias judgements for each study are presented in Supplementary Figure 1. One study was rated low risk across all domains [27]. Five studies were rated high risk for the randomisation process [28–32]. This was reportedly due to allocation being based on availability of beds on each ward [28], ward admitted to [29], or based on the date of admission (e.g., rotating every four weeks or different timeframes used for the control and intervention groups) [30–32]. All studies were considered low risk for deviations from the intended interventions. For missing outcome data, the following outcomes were affected by a large amount of missing data: sit-to-stand and balance-sway area (not included in pooled analyses). Measurement of the outcome was low in two studies and unclear in four studies, due to lack of blinding of outcome assessors. Selection of the reported result was low for three studies, unclear for three studies due to no trial registration or protocol to compare against.

### Primary outcomes: physical activity interventions versus usual care

Some primary outcomes were not included in pooled analyses as they were only available in one study. Schwenk et al. found significant improvements in the intervention group for leg press (kg), abductor (kg), and balance (sway area, cm^2^), but no significant difference was found for gait (cadence: steps/min), handgrip (kg), and hierarchical assessment of balance and mobility (HABAM) [28]. No significant difference was also found on the Alusti test [31], functional independent measure (FIM) [27], and the Alzheimer’s Disease Cooperative Study-ADL (ADCS-ADL) [30].

#### Strength: time (seconds) to complete five sit-to-stand repetitions at end of intervention/discharge from hospital (change from baseline)

**Fig. 2:**
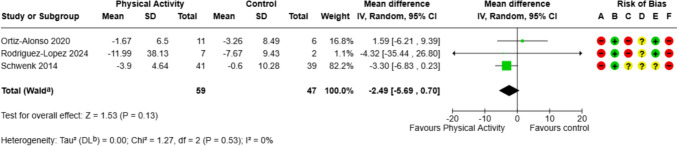
Strength: time to complete five sit-to-stand repetitions at end of intervention/discharge from hospital (change from baseline). CI calculated by Wald-type method, Tau^2^ calculated by DerSimonian and Laird method. Standard deviation of change scores for Schwenk 2014 imputed. Risk-of-bias legend: **A** randomisation process, **B** deviations from intended interventions, **C** missing outcome data, **D** measurement of the outcome, **E** selection of the reported result, and **F** overall bias

There was no significant difference found in the time to perform sit-to-stand repetitions between the intervention and control groups (three studies; mean difference − 2.49, 95% CI − 5.69 to 0.70) (Fig. 2).

#### Functional ability: independence in basic ADLs at end of intervention/discharge from hospital (change from baseline scores at admission)

**Fig. 3:**
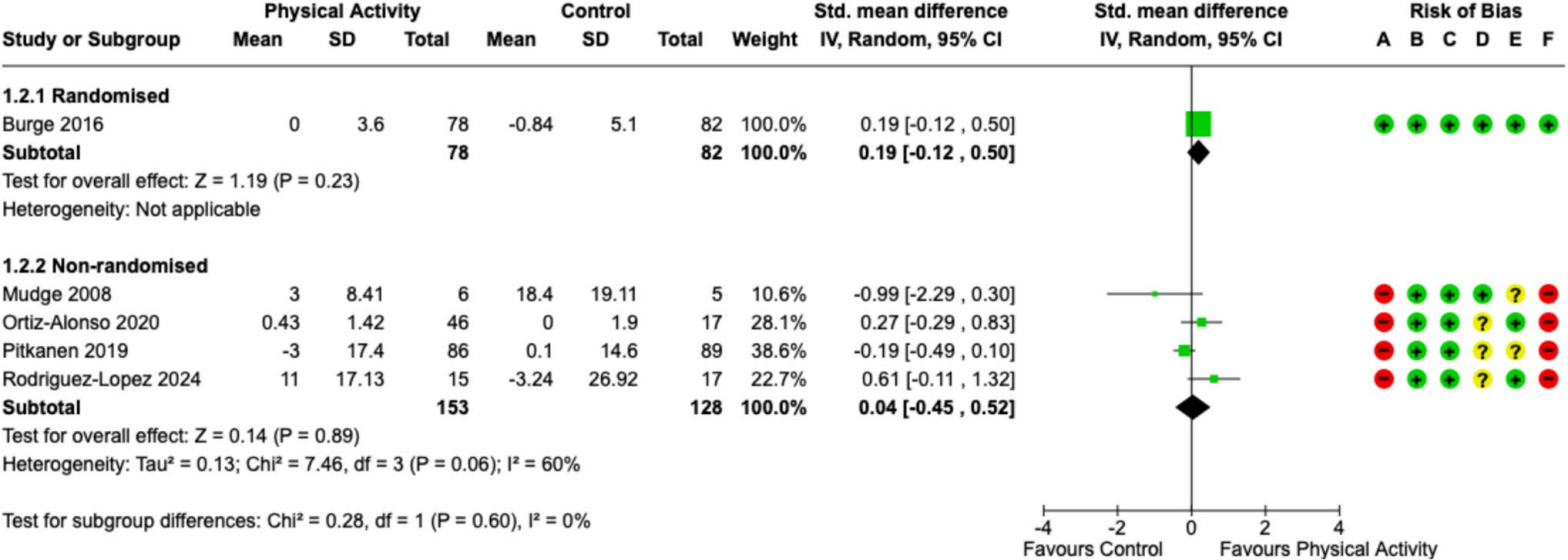
Functional ability: independence in basic activities of daily living at end of intervention/discharge from hospital (change from baseline). CI calculated by Wald-type method, Tau^2^ calculated by DerSimonian and Laird method. Standard deviation of change scores for Burge 2016 imputed. Risk-of-bias legend: **A** randomisation process, **B** deviations from intended interventions, **C** missing outcome data, **D** measurement of the outcome, **E** selection of the reported result, and **F** overall bias

Five studies measured independence in basic ADLs; three studies used the Barthel index, one used the modified version of the Barthel, and one used the Katz index. In the RCT, there was no significant difference between the intervention and control groups. From the non-randomised evidence, there was also no significant difference found (four studies; standardised mean difference 0.04, 95% CI − 0.45 to 0.52, moderate heterogeneity) (Fig. 3).

#### Functional ability: independence in basic ADLs at discharge from hospital (change from baseline scores at pre-admission)

There was no significant difference found between the intervention and control groups on the Katz index at discharge when using change from pre-admission scores (two studies; mean difference 0.38, 95% CI − 0.35 to 1.12) (Supplementary Figure 2).

#### Functional ability: independence in basic ADLs at 3 months post-discharge

There was a significant difference on the Katz index at 3 months when using change from pre-admission scores, in favour of physical activity intervention (two studies; mean difference 1.27, 95% CI 0.36 to 2.18), but there was no significant difference found when using change from admission scores (two studies; mean difference 0.27, 95% CI − 1.40 to 1.94, moderate heterogeneity) (Supplementary Figure 3).

#### Independent walking ability: functional ambulatory classification (FAC) at discharge (change from baseline)

There was no significant difference found between the intervention and control groups on the modified FAC at discharge when using change from pre-admission scores (two studies; mean difference 0.51, 95% CI − 0.17 to 1.18, moderate heterogeneity) and change from admission scores (two studies; mean difference 0.21, 95% CI − 0.32 to 0.75) (Supplementary Figure 4).

#### Independent walking ability: functional ambulatory classification (FAC) at 3 months post-discharge (change from baseline)

There was no significant difference found between the intervention and control groups on the modified FAC at 3 months when using change from pre-admission scores (two studies; mean difference 0.66, 95% CI − 0.08 to 1.39) and change from admission scores (two studies; mean difference 0.21, 95% CI − 0.68 to 1.10) (Supplementary Figure 5).

#### Gait speed: distance (cm/s) at discharge from hospital (change from baseline)

Four studies measured gait speed, but as studies used different walking distances, data were converted to cm/second. There was no significant difference in gait speed between the intervention and control groups (four studies; mean difference 2.83, 95% CI − 2.77 to 8.42) (Supplementary Figure 6).

#### Balance: Short Physical Performance Battery (SPPB) subtest at discharge from hospital (change from baseline)

There was no significant difference in balance between the intervention and control groups (two studies; mean difference 0.27, 95% CI − 0.33 to 0.88), moderate heterogeneity (Supplementary Figure 7).

### Secondary outcomes: physical activity interventions versus usual care

#### Falls: number of fallers (in hospital or within 3 months)

One study recorded inpatient falls, and two studies recorded falls post-discharge (within 3 months). There was no significant difference in the number of people having falls between the intervention and control groups (three studies; odds ratio 1.12, 95% CI 0.32 to 3.89) (Supplementary Figure 8).

#### Readmission to hospital within 3 months

There was no significant difference between the intervention and control groups (two studies; odds ratio 1.42, 95% CI 0.14 to 14.01, high heterogeneity) (Supplementary Figure 9).

#### Length of stay (days)

There was no significant difference between the intervention and control groups (three studies; mean difference − 1.10, 95% CI − 3.14 to 0.93) (Supplementary Figure 10).

#### Incident delirium

There was no significant difference between the intervention and control groups (two studies; odds ratio 0.67, 95% CI 0.23 to 1.95) (Supplementary Figure 11).

### GRADE assessment

See Supplementary Tables 3 and 4 for summary of findings. We downgraded based on high risk of bias (non-randomisation or quasi-randomisation), presence of statistical heterogeneity, wide confidence intervals, and small sample sizes/small number of events. Overall, the certainty of evidence is very low or low.

## Discussion

Overall, there was limited available evidence specifically in the context of dementia and the certainty of that evidence is very low or low. In pooled analyses, we did not find any differences between the intervention and control groups at discharge, on outcomes such as ADLs, sit-to-stand repetitions, gait speed, and balance. At 3 months post-discharge, there was less decline in independence in basic ADLs in the physical activity group when using change from pre-admission scores, but not when using change from admission scores. There were no differences on other outcomes at 3 months. However, these conclusions are limited by the low certainty of evidence, so results might be expected to change if additional high-quality studies are conducted.

Despite the predominantly non-significant findings across meta-analyses, visual inspection of the forest plots suggests a trend favouring physical activity intervention in most analyses. The results from this review should be interpreted in the context of various factors. First, all studies included usual care as the control group, which often involved being seen by the therapy team and mobilisation. These studies may not reflect the reality of everyday care, where many people living with dementia get minimal rehabilitation [21, 35] and where physical activity interventions could be particularly beneficial. The different ward settings meant that usual care differed across studies; for example, the detailed description of care on the ACE unit in one study suggested that they took a more proactive approach to daily mobilisation than the acute psychogeriatric wards. Another factor is the frequency and dosage of interventions may not have been enough, for example, in one study, the mean number of days the intervention was received was less than 3 [32], which may have been due to a short length of stay or adherence. Where adherence was reported, it was below the mean rate of 70% reported in a recent review of adherence to exercise in mild cognitive impairment and dementia [36]. Finally, the outcome measures most proximal to deconditioning are strength-based and apart from sit-to-stand, these were only available in one study [28], which reported significant improvements. Using total score of a basic ADL scale as an outcome measure is likely not a sensitive metric, since we would expect some ADLs to be more affected than others by physical activity. This was demonstrated in the study by Burge et al. [33], where item-level analysis showed that different ADLs worsened across the intervention and control groups: scores in bladder and bowel control dropped in the intervention group, whereas in the control group, there were declines in stairs, mobility, toilet use, and transfer. There was clinical heterogeneity across studies, including types of wards and interventions (type of physical activity and multi/single component). Despite this, we decided to pool multicomponent and single component interventions, as the additional components included music, education, and cognitive stimulation therapy which are unlikely to be driving changes in physical functioning outcomes.

We used change from baseline data, but baseline assessment was not always conducted on the first day of admission, which is often not possible when written consent is needed prior to assessment. Ideally, the pre-hospital status is used as a baseline, since people can decline within the first day of admission [37]. Pre-admission scores were only available in two studies for two outcomes (derived through retrospective interview), and these scores differed to those at admission (decline in ADLs had already occurred for many participants). The choice of baseline (pre-admission/admission) affects results, as illustrated in the analysis of basic ADLs at 3 months. There was less decline in the intervention group at 3 months; however, this was only when using change from pre-admission scores. A delayed effect could be due to continued physical activity following discharge, since one of the two studies included in this analysis included monthly telephone counselling to encourage participants to continue the same exercise routine at home. However, this effect is unlikely given there was no difference at discharge and there was no difference at 3 months when change from admission scores were used. The difference in results likely arises from the small difference in sample size between analyses (three additional participants in the change from pre-admission analysis) and changes in scores between pre-admission and admission.

Our conclusions are similar to other reviews conducted which are not dementia specific; there is uncertainty of the effect of physical activity interventions above usual care [15, 38]. However, the certainty of evidence is higher regarding no higher risk of falls [15]. This review identified additional hospital-based interventions that could help prevent deconditioning, but the studies did not meet the full inclusion criteria. These include training programmes for staff [39], involving family members in goal setting and care planning [40], training volunteers to support nutrition and hydration, hearing/visual aids, activities, and orientation [41], and virtual reality interventions [42].

Strengths of this review include following best practice guidance, including assessing the certainty of evidence using GRADE and contacting authors to obtain unpublished data. Limitations were exclusion of studies written in a non-English language and the small number of eligible studies which precluded any subgroup and sensitivity analyses or assessment of publication bias. The small sample sizes in analyses also mean that they are likely underpowered. There were additional eligible studies (where a subsample of the participants had dementia), but authors did not provide a dataset for inclusion. Using change from baseline data was limited by the timing of the baseline assessment and also meant that sample sizes were reduced where participants did not have scores at both timepoints.

Although there is insufficient high-quality evidence to recommend which types of physical activity interventions could be most beneficial for hospitalised people living with dementia, we should ensure that regular physical activity is encouraged in hospital, since there is evidence of the harms of deconditioning [5, 43]. A systematic review found that goal setting and feedback are independently associated with increases in physical activity in hospital [44], which are key components of rehabilitation. Resistance training is recommended in older adults for building muscle strength and reducing deconditioning [45, 46]. Rehabilitation should be tailored and person-centred; generalisations about rehabilitation potential should not be made based on the presence of a dementia diagnosis. There is no evidence suggesting that physical activity interventions increase falls, which is important, since this can often be a reason for restricting movement. However, the interventions in the included studies were typically supervised, which is not always feasible in current practice. Risk assessments typically focus on falls risk but should also consider the harm of leaving people in bed. Ensuring patients are wearing their own footwear, well-fitting trousers and glasses/hearing aids (where needed) are also important to minimise risk. Regular mobilisation and exercises should not be limited to the context of formal rehabilitation but also facilitated outside these sessions by all members of the multidisciplinary team.

More research is needed including people living with dementia in the hospital setting. Future research should ensure the description of the intervention is detailed enough for replication, following the UK Medical Research Council’s guidance for complex interventions [47], include data on the amount of intervention received (digital measures of activity could be considered), deviations from protocol, rates of decline, and severity of dementia. Qualitative research with people living with dementia is also an important area for future research to explore any potential barriers and to understand the types of activities they would like to be offered in hospital.

Overall, due to the limited available evidence, there is uncertainty about the effect of physical activity interventions above usual care. More high-quality research is needed to improve outcomes for people living with dementia in hospital.

## Supplementary Information

Below is the link to the electronic supplementary material.

Supplementary file1 (DOCX 717 KB)

## Data Availability

This is a review of previously published studies. Some of the included data are available in the publications of included studies. We obtained unpublished data for participants with dementia for three studies for inclusion in the review. These datasets can be requested from the authors of the included studies.
