# Supplementary material for: Physical activity interventions for hospitalised people living with dementia: systematic review and meta-analysis
Source: Eur Geriatr Med. 2025 Sep 16;16(6):2021–31. doi: 10.1007/s41999-025-01304-x (PMC12743704; doi:10.1007/s41999-025-01304-x)
Supplement: Supplementary file 1 — Supplementary file1 (DOCX 717 KB) [file 41999_2025_1304_MOESM1_ESM.docx]

**Supplementary materials**

# **Supplementary methods 1: Search strategies**

| **Medline** | |
| --- | --- |
| 1 | exp Dementia/ |
| 2 | dement*.tw. |
| 3 | (alzheimer* or alzeimer*).tw. |
| 4 | neurocognitive disorder.tw. |
| 5 | 1 or 2 or 3 or 4 |
| 6 | exp Exercise/ or exp Exercise Therapy/ |
| 7 | exercis*.tw. |
| 8 | physical activit*.tw. |
| 9 | physical therapy.tw. |
| 10 | gym*.tw. |
| 11 | walk*.tw. |
| 12 | mobilis*.tw. |
| 13 | ambulat*.tw. |
| 14 | rehabilitat*.tw. |
| 15 | exp Walking/ |
| 16 | 6 or 7 or 8 or 9 or 10 or 11 or 12 or 13 or 14 or 15 |
| 17 | decondition*.tw. |
| 18 | exp "Activities of Daily Living"/ |
| 19 | activities of daily living.tw. |
| 20 | ADLs.tw. |
| 21 | exp Muscle Strength/ |
| 22 | strength.tw. |
| 23 | muscle mass.tw. |
| 24 | exp Frail Elderly/ or exp Frailty/ |
| 25 | frail*.tw. |
| 26 | physical function.tw. |
| 27 | aerobic capacity.tw. |
| 28 | exercise capacity.tw. |
| 29 | exp Exercise Test/ |
| 30 | pj paralysis.tw. |
| 31 | functional dependenc*.tw. |
| 32 | functional decline.tw. |
| 33 | functional disability.tw. |
| 34 | functional ability.tw. |
| 35 | functional status.tw. |
| 36 | functional deterioration.tw. |
| 37 | fall*.tw. |
| 38 | balance.tw. |
| 39 | exp Postural Balance/ |
| 40 | 17 or 18 or 19 or 20 or 21 or 22 or 23 or 24 or 25 or 26 or 27 or 28 or 29 or 30 or 31 or 32 or 33 or 34 or 35 or 36 or 37 or 38 or 39 |
| 41 | exp Hospitals/ |
| 42 | hospital*.tw. |
| 43 | 84 or 85 |
| 44 | 48 and 59 and 83 and 86 |

| **APA PsycInfo** | |
| --- | --- |
| 1 | exp Dementia/ |
| 2 | dement*.tw. |
| 3 | (alzheimer* or alzeimer*).tw. |
| 4 | neurocognitive disorder.tw. |
| 5 | 1 or 2 or 3 or 4 |
| 6 | exp Exercise/ or exp Exercise Therapy/ |
| 7 | exercis*.tw. |
| 8 | physical activit*.tw. |
| 9 | physical therapy.tw. |
| 10 | gym*.tw. |
| 11 | walk*.tw. |
| 12 | mobilis*.tw. |
| 13 | ambulat*.tw. |
| 14 | rehabilitat*.tw. |
| 15 | exp Walking/ |
| 16 | 6 or 7 or 8 or 9 or 10 or 11 or 12 or 13 or 14 or 15 |
| 17 | decondition*.tw. |
| 18 | exp "Activities of Daily Living"/ |
| 19 | activities of daily living.tw. |
| 20 | ADLs.tw. |
| 21 | exp Physical Strength/ |
| 22 | strength.tw. |
| 23 | muscle mass.tw. |
| 24 | frail*.tw. |
| 25 | physical function.tw. |
| 26 | aerobic capacity.tw. |
| 27 | exercise capacity.tw. |
| 28 | exp Physical fitness/ |
| 29 | pj paralysis.tw. |
| 30 | functional dependenc*.tw. |
| 31 | functional decline.tw. |
| 32 | functional disability.tw. |
| 33 | functional ability.tw. |
| 34 | functional status.tw. |
| 35 | functional deterioration.tw. |
| 36 | fall*.tw. |
| 37 | balance.tw. |
| 38 | exp Falls/ |
| 39 | exp Hospitals/ |
| 40 | hospital*.tw. |
| 41 | 39 or 40 |
| 42 | 17 or 18 or 19 or 20 or 21 or 22 or 23 or 24 or 25 or 26 or 27 or 28 or 29 or 30 or 31 or 32 or 33 or 34 or 35 or 36 or 37 or 38 |
| 43 | 5 and 16 and 41 and 42 |

| **Embase** | |
| --- | --- |
| 1 | exp Dementia/ |
| 2 | dement*.tw. |
| 3 | (alzheimer* or alzeimer*).tw. |
| 4 | neurocognitive disorder.tw. |
| 5 | 1 or 2 or 3 or 4 |
| 6 | exp Exercise/ or exp Exercise Therapy/ |
| 7 | exercis*.tw. |
| 8 | physical activit*.tw. |
| 9 | physical therapy.tw. |
| 10 | gym*.tw. |
| 11 | walk*.tw. |
| 12 | mobilis*.tw. |
| 13 | ambulat*.tw. |
| 14 | rehabilitat*.tw. |
| 15 | exp Walking/ |
| 16 | 6 or 7 or 8 or 9 or 10 or 11 or 12 or 13 or 14 or 15 |
| 17 | decondition*.tw. |
| 18 | exp "Activities of Daily Living"/ |
| 19 | activities of daily living.tw. |
| 20 | ADLs.tw. |
| 21 | exp Muscle Strength/ |
| 22 | strength.tw. |
| 23 | muscle mass.tw. |
| 24 | exp Frail Elderly/ or exp Frailty/ |
| 25 | frail*.tw. |
| 26 | physical function.tw. |
| 27 | aerobic capacity.tw. |
| 28 | exercise capacity.tw. |
| 29 | exp Exercise Test/ |
| 30 | pj paralysis.tw. |
| 31 | functional dependenc*.tw. |
| 32 | functional decline.tw. |
| 33 | functional disability.tw. |
| 34 | functional ability.tw. |
| 35 | functional status.tw. |
| 36 | functional deterioration.tw. |
| 37 | fall*.tw. |
| 38 | balance.tw. |
| 39 | exp Postural Balance/ |
| 40 | 17 or 18 or 19 or 20 or 21 or 22 or 23 or 24 or 25 or 26 or 27 or 28 or 29 or 30 or 31 or 32 or 33 or 34 or 35 or 36 or 37 or 38 or 39 |
| 41 | exp Hospitals/ |
| 42 | hospital*.tw. |
| 43 | 41 or 42 |

| **CENTRAL** | |
| --- | --- |
| #1 | dement* |
| #2 | alzheimer* |
| #3 | MeSH descriptor: [Dementia] explode all trees |
| #4 | #1 OR #2 OR #3 |
| #5 | MeSH descriptor: [Exercise] explode all trees |
| #6 | (exercis*):ti,ab,kw |
| #7 | (physical NEXT activit*):ti,ab,kw |
| #8 | (walk*):ti,ab,kw |
| #9 | ("physical therapy"):ti,ab,kw |
| #10 | (mobilis*):ti,ab,kw |
| #11 | #5 OR #6 OR #7 OR #8 OR #9 OR #10 |
| #12 | (hospital*):ti,ab,kw |
| #13 | #4 AND #11 AND #12 in Cochrane Protocols, Trials |

| **CINAHL** | |
| --- | --- |
| S46 | S5 AND S17 AND S42 AND S45 |
| S45 | S43 OR S44 |
| S44 | AB hospital* |
| S43 | (MH "Hospitals+") |
| S42 | S18 OR S19 OR S20 OR S21 OR S22 OR S23 OR S24 OR S25 OR S26 OR S27 OR S28 OR S29 OR S30 OR S31 OR S32 OR S33 OR S34 OR S35 OR S36 OR S37 OR S38 OR S39 OR S40 OR S41 |
| S41 | (MH "Balance, Postural+") |
| S40 | AB balance |
| S39 | AB fall* |
| S38 | AB "functional deterioration" |
| S37 | AB "functional status" |
| S36 | AB "functional ability" |
| S35 | AB "functional disability" |
| S34 | AB "functional decline" |
| S33 | AB "functional dependenc*" |
| S32 | AB "pj paralysis" |
| S31 | (MH "Exercise Test+") |
| S30 | AB "exercise capacity" |
| S29 | AB "aerobic capacity" |
| S28 | AB "physical function" |
| S27 | AB frail* |
| S26 | (MM "Frail Elderly") |
| S25 | (MM "Frailty Syndrome") |
| S24 | AB "muscle mass" |
| S23 | AB strength |
| S22 | (MH "Muscle Strength+") |
| S21 | AB ADLs |
| S20 | AB "activities of daily living" |
| S19 | (MH "Activities of Daily Living+") |
| S18 | AB decondition* |
| S17 | S6 OR S7 OR S8 OR S9 OR S10 OR S11 OR S12 OR S13 OR S14 OR S15 OR S16 |
| S16 | (MH "Walking+") |
| S15 | AB rehabilitat* |
| S14 | AB ambulat* |
| S13 | AB mobilis* |
| S12 | AB walk* |
| S11 | AB gym* |
| S10 | AB "physical therapy" |
| S9 | AB physical activit* |
| S8 | AB exercis* |
| S7 | (MH "Therapeutic Exercise+") |
| S6 | (MH "Exercise+") |
| S5 | S1 OR S2 OR S3 OR S4 |
| S4 | AB "neurocognitive disorder" |
| S3 | AB alzheimer* |
| S2 | AB dement* |
| S1 | (MH "Dementia+") |

# **Supplementary methods 2: Inclusion Criteria**

Participants/population

- Hospitalised patients (≥18 years)
- Documented diagnosis of dementia (all causes/aetiologies will be included). Diagnosis of dementia can be according to any diagnostic criteria and details on diagnosis not needed for inclusion. Exclude studies which do not state a diagnosis of dementia, only a cognitive screening test score, due to limitations of cognitive tests (people can score low for other reasons than cognition, e.g., low education, ethnic minorities, physical impairments)
- In studies including participants with dementia along with those without, data need to be provided separately for those with dementia (or authors need to be willing to share dataset) to be included.
- Exclude patients admitted to hospital due to a stroke or traumatic brain injury.

Context

- Patients with a diagnosis of dementia who are admitted via acute hospital.
- Exclude studies where patients are admitted to a high dependency/critical care ward or neurorehabilitation for stroke/TBI.

Intervention(s), exposure(s)

- Any intervention taking place during hospitalisation involving physical activity, mobilisation or exercise is eligible. Participants in the intervention group need to undertake more physical activity than the control group
- The study does not have to explicitly state the aim of the intervention is to prevent or treat deconditioning, but the intervention should increase physical activity during the period that the patient is hospitalised.

Comparator(s)/control

- Usual care during hospital admission or another activity involving less physical activity (e.g., a sit-down activity).

Outcome measures

- Main outcomes
  - Deconditioning is a multifaceted concept with no standardised outcomes. The main outcomes will include direct or proxy measures of hospital acquired deconditioning, including strength (strength and muscle mass), aerobic or exercise capacity, functional ability (mobility, balance, basic and instrumental ADLs).
  - Eligible studies must include at least one of the above main outcomes.
  - We will include measures that are performance based, clinician reported, or caregiver/patient reported.
- Secondary outcomes
  - Safety (adverse events), for example pressure ulcers/bed sores, falls, delirium.
  - Frailty, length of stay, discharge destination, changes to package of care, mortality, readmission rates, quality of life and mood

Study type

- Both randomised and non-randomised controlled studies are eligible

# **Supplementary Table 1: PRISMA checklist**

| **Section and Topic** | **Item #** | **Checklist item** | **Location where item is reported** |
| --- | --- | --- | --- |
| **TITLE** | | |  |
| Title | 1 | Identify the report as a systematic review. | 1 |
| **ABSTRACT** | | |  |
| Abstract | 2 | See the PRISMA 2020 for Abstracts checklist. | 2 |
| **INTRODUCTION** | | |  |
| Rationale | 3 | Describe the rationale for the review in the context of existing knowledge. | 3 |
| Objectives | 4 | Provide an explicit statement of the objective(s) or question(s) the review addresses. | 3 |
| **METHODS** | | |  |
| Eligibility criteria | 5 | Specify the inclusion and exclusion criteria for the review and how studies were grouped for the syntheses. | 3-4 and suppl materials |
| Information sources | 6 | Specify all databases, registers, websites, organisations, reference lists and other sources searched or consulted to identify studies. Specify the date when each source was last searched or consulted. | 3-4 |
| Search strategy | 7 | Present the full search strategies for all databases, registers and websites, including any filters and limits used. | Suppl materials |
| Selection process | 8 | Specify the methods used to decide whether a study met the inclusion criteria of the review, including how many reviewers screened each record and each report retrieved, whether they worked independently, and if applicable, details of automation tools used in the process. | 4 |
| Data collection process | 9 | Specify the methods used to collect data from reports, including how many reviewers collected data from each report, whether they worked independently, any processes for obtaining or confirming data from study investigators, and if applicable, details of automation tools used in the process. | 4 |
| Data items | 10a | List and define all outcomes for which data were sought. Specify whether all results that were compatible with each outcome domain in each study were sought (e.g. for all measures, time points, analyses), and if not, the methods used to decide which results to collect. | 4 and suppl materials |
|  | 10b | List and define all other variables for which data were sought (e.g. participant and intervention characteristics, funding sources). Describe any assumptions made about any missing or unclear information. | 4 |
| Study risk of bias assessment | 11 | Specify the methods used to assess risk of bias in the included studies, including details of the tool(s) used, how many reviewers assessed each study and whether they worked independently, and if applicable, details of automation tools used in the process. | 4 |
| Effect measures | 12 | Specify for each outcome the effect measure(s) (e.g. risk ratio, mean difference) used in the synthesis or presentation of results. | 4 |
| Synthesis methods | 13a | Describe the processes used to decide which studies were eligible for each synthesis (e.g. tabulating the study intervention characteristics and comparing against the planned groups for each synthesis (item #5)). | 4 |
|  | 13b | Describe any methods required to prepare the data for presentation or synthesis, such as handling of missing summary statistics, or data conversions. | 4 |
|  | 13c | Describe any methods used to tabulate or visually display results of individual studies and syntheses. | 5 |
|  | 13d | Describe any methods used to synthesize results and provide a rationale for the choice(s). If meta-analysis was performed, describe the model(s), method(s) to identify the presence and extent of statistical heterogeneity, and software package(s) used. | 4 |
|  | 13e | Describe any methods used to explore possible causes of heterogeneity among study results (e.g. subgroup analysis, meta-regression). | NA |
|  | 13f | Describe any sensitivity analyses conducted to assess robustness of the synthesized results. | NA |
| Reporting bias assessment | 14 | Describe any methods used to assess risk of bias due to missing results in a synthesis (arising from reporting biases). | NA |
| Certainty assessment | 15 | Describe any methods used to assess certainty (or confidence) in the body of evidence for an outcome. | 4 |
| **RESULTS** | | |  |
| Study selection | 16a | Describe the results of the search and selection process, from the number of records identified in the search to the number of studies included in the review, ideally using a flow diagram. | Figure 1 |
|  | 16b | Cite studies that might appear to meet the inclusion criteria, but which were excluded, and explain why they were excluded. | Suppl materials |
| Study characteristics | 17 | Cite each included study and present its characteristics. | 5 and table 1 |
| Risk of bias in studies | 18 | Present assessments of risk of bias for each included study. | Suppl materials |
| Results of individual studies | 19 | For all outcomes, present, for each study: (a) summary statistics for each group (where appropriate) and (b) an effect estimate and its precision (e.g. confidence/credible interval), ideally using structured tables or plots. | 6-7 |
| Results of syntheses | 20a | For each synthesis, briefly summarise the characteristics and risk of bias among contributing studies. | 6-7 |
|  | 20b | Present results of all statistical syntheses conducted. If meta-analysis was done, present for each the summary estimate and its precision (e.g. confidence/credible interval) and measures of statistical heterogeneity. If comparing groups, describe the direction of the effect. | 6-7 |
|  | 20c | Present results of all investigations of possible causes of heterogeneity among study results. | NA |
|  | 20d | Present results of all sensitivity analyses conducted to assess the robustness of the synthesized results. | NA |
| Reporting biases | 21 | Present assessments of risk of bias due to missing results (arising from reporting biases) for each synthesis assessed. | NA |
| Certainty of evidence | 22 | Present assessments of certainty (or confidence) in the body of evidence for each outcome assessed. | Table 2 |
| **DISCUSSION** | | |  |
| Discussion | 23a | Provide a general interpretation of the results in the context of other evidence. | 7-8 |
|  | 23b | Discuss any limitations of the evidence included in the review. | 8 |
|  | 23c | Discuss any limitations of the review processes used. | 8 |
|  | 23d | Discuss implications of the results for practice, policy, and future research. | 8-9 |
| **OTHER INFORMATION** | | |  |
| Registration and protocol | 24a | Provide registration information for the review, including register name and registration number, or state that the review was not registered. | 3 |
|  | 24b | Indicate where the review protocol can be accessed, or state that a protocol was not prepared. | 3 |
|  | 24c | Describe and explain any amendments to information provided at registration or in the protocol. | NA |
| Support | 25 | Describe sources of financial or non-financial support for the review, and the role of the funders or sponsors in the review. | 9 |
| Competing interests | 26 | Declare any competing interests of review authors. | NA |
| Availability of data, code and other materials | 27 | Report which of the following are publicly available and where they can be found: template data collection forms; data extracted from included studies; data used for all analyses; analytic code; any other materials used in the review. | NA |

# **Supplementary Table 2: Relevant excluded studies, with reasons**

| **Study ID** | **Title** | **Reason for Exclusion** |
| --- | --- | --- |
| Blair 2018 | The “Golden Angels”: effects of trained volunteers on specialling and readmission rates for people with dementia and delirium in rural hospitals | Wrong population - Not all participants had a diagnosis of dementia (some had delirium or risk factors for delirium).  Incorrect outcome measures |
| Chang 2020 | Effect of Resistance Exercise on Depression in Mild Alzheimer Disease Patients With Sarcopenia | Setting – described as nursing hospital, which is likely to be equivalent of a care home. Author emailed for clarification but no reply |
| Huusko 2000 | Randomised, clinically controlled trial of intensive geriatric rehabilitation in patients with hip fracture: subgroup analysis of patients with dementia | Incorrect outcome measures |
| Jeffs 2013 | An enhanced exercise and cognitive programme does not appear to reduce incident delirium in hospitalised patients: a randomised controlled trial | Data not separate for those with dementia |
| Kampragkou 2017 | Effects of a 12-week aerobic exercise program combined with music therapy and memory exercises on cognitive and functional ability in people with middle type of Alzheimer’s disease | Setting – participants are described as being “permanently hospitalised” |
| Stenvall 2007 | A multidisciplinary, multifactorial intervention program reduces postoperative falls and injuries after femoral neck fracture | Intervention and control groups both included exercise and rehabilitation – intervention group didn’t receive more. Incorrect outcome measures |
| Stenvall 2012 | A multidisciplinary intervention program improved the outcome after hip fracture for people with dementia—Subgroup analyses of a randomized controlled trial | Outcomes measure only available at 4 months |
| Yun 2021 | A Simple Bedside Exercise Method to Enhance Lower Limb Muscle Strength in Moderate Alzheimer’s Disease Patients with Sarcopenia | Setting – described as nursing hospital, which is likely to be equivalent of a care home. Author emailed for clarification but no reply |

**Supplementary Table 3: Summary of Findings for Primary Outcomes**

| Outcomes | **Anticipated absolute effects^*^** (95% CI) | | № of participants (studies) | Certainty of the evidence (GRADE) | Comments |
| --- | --- | --- | --- | --- | --- |
|  | **Risk with Usual Care** | **Risk with Physical Activity** |  |  |  |
| **5 Chair Sit-to-Stand** assessed with: seconds  Measured at the end of intervention or discharge | Mean change score in CG ranged -7.67 to -0.60 | MD 2.49 **lower** (5.69 lower to 0.70 higher) | 106 (3 non-randomised studies) | ⨁◯◯◯ Very low^a,e^ | The unweighted mean change from baseline for the intervention group was -5.85 and for the control group -3.84 |
| **Gait speed** assessed with: distance (cm/sec)  Measured at the end of intervention or discharge | Mean change score in CG ranged -5.39 to 16.10 | MD **2.83 higher** (2.77 lower to 8.42 higher) | 197  (4 non-randomised studies) | ⨁◯◯◯ Very low^a,e^ |  |
| **Balance** assessed with: SPPB subtest from: 0 to 4  Measured at discharge | Mean change score in CG ranged -0.24 to 0.07 | MD **0.27 higher** (0.33 lower to 0.88 higher) | 88 (2 non-randomised studies) | ⨁◯◯◯ Very low^b,c,e^ |  |
| **Balance: Sway area** assessed with: sq cm  Measured at the end of intervention | Mean change score in CG was -0.10 | Mean change score in IG was -0.40 | 81 (1 non-randomised study) | ⨁◯◯◯ Very low^c,e^ | % change data were reported: IG: -5.9± 8.5, CG: -0.8±10.4 |
| **Basic ADLs** - Randomised assessed with: Barthel Index from: 0 to 100  Measured at the end of intervention | Mean change score was -0.84 | SMD **0.19 higher** (0.12 lower to 0.50 higher) | 160 (1 RCT) | ⨁⨁◯◯ Low^c^ |  |
| **Basic ADLs** - Non-randomised assessed with: Barthel Index, Katz index from: 0 to 6  Measured at the end of intervention or discharge | Mean change score in CG ranged -3.24 to 18.40 | SMD **0.04 higher** (0.45 lower to 0.52 higher) | 281 (4 non-randomised studies) | ⨁◯◯◯ Very low^b,e^ |  |
| **Basic ADLs** (change from pre-admission) assessed with: Katz index from 0 to 6  Measured at discharge | Mean change score in CG ranged -1.35 to -1.94 | **MD 0.38 higher**  (0.35 lower to 1.12 higher) | 96 (2 non-randomised studies) | ⨁⨁◯◯ Low^e^ |  |
| **ADLs** assessed with: ADCS-ADL from 0 to 78  Measured at discharge | Mean change score in CG was -10.5 | MD **0.70 lower** (5.10 lower to 3.70 higher) | 175  (1 non-randomised study) | ⨁◯◯◯ Very low^c,e^ |  |
| **Motor and cognitive ADLs** assessed with functional independence measure from 18 to 126  Measured straight after intervention and two weeks after | Mean change score in CG was -3.34 | Mean change score in IG was -0.69 | 160 (1 RCT) | ⨁⨁◯◯ Low^c^ | 2 weeks after intervention: Mean change score was -6.38 in CG, -3.84 in IG |
| **Basic ADLs** (change from pre-admission) assessed with: Katz index from 0 to 6  Measured at 3 months | Mean change score in CG ranged -1.06 to -1.93 | MD **1.27 higher**  (0.36 higher to 2.18 higher) | 82 (2 non-randomised studies) | ⨁⨁◯◯ Low^e^ | Change from admission also available (n=79) |
| **Independent walking ability** (change from pre-admission) assessed with: Functional Ambulatory Capacity modified from 0 to 4  Measured at discharge | Mean change score in CG ranged -1.06 to -0.88 | MD **0.51 higher**  (0.17 lower to 1.18 higher) | 96  (2 non-randomised studies) | ⨁⨁◯◯ Low^e^ | Change from admission also available (n=94) |
| **Independent walking ability** (change from pre-admission) assessed with: Functional Ambulatory Capacity modified from 0 to 4  Measured at 3 months | Mean change score in CG ranged -0.81 to -1.46 | MD **0.66 higher**  (0.08 lower to 1.39 higher) | 81  (2 non-randomised studies) | ⨁⨁◯◯ Low^e^ | Change from admission also available (n=78) |
| **Gait cadence** assessed with steps/min  Measured at the end of intervention | Mean change score in CG was 9.9 | Mean change score in IG was 12.9 | 109 (1 non-randomised study) | ⨁◯◯◯ Very low^c,e^ | % change data were reported: IG +16.8 ±26.0, CG: +12.8 ±26.2 |
| **Leg Strength** assessed with leg press 1-RM, kg  Measured at the end of intervention | Mean change score in CG was 5.2 | Mean change score in IG was 40.3 | 125 (1 non-randomised study) | ⨁◯◯◯ Very low^c,e^ | % change data were reported: IG +51.9 ±42.3, CG: +13.5 ±51.8 |
| **Abductor strength** assessed with 1-RM, kg  Measured at the end of intervention | Mean change score in CG was 2.8 | Mean change score in IG was 17.4 | 111 (1 non-randomised study) | ⨁◯◯◯ Very low^c,e^ | % change data were reported: IG: +21.0 ±25.4, CG: +10.6 ±38.2 |
| **Handgrip strength** assessed with kg  Measured at the end of intervention | Mean change score in CG was 0.5 | Mean change score in IG was 0.4 | 125 (1 non-randomised study) | ⨁◯◯◯ Very low^c,e^ | % change data were reported: IG: +6.6± 43.9, CG: +5.7±21.0 |
| **Balance and Mobility** assessed with HABAM from 0 to 65  Measured at the end of intervention | Mean change score in CG was 5.5 | Mean change score in IG was 8.1 | 123 (1 non-randomised study) | ⨁◯◯◯ Very low^c,e^ | % change data were reported: IG: +32.6± 48.7, CG: +22.1±32.9 |
| **Functional assessment**  assessed with Alusti test from 0 to 100  Measured at discharge | Mean change score in CG was -0.67 | MD **1.12 higher** (7.06 lower to 9.30 higher) | 23 (1 non-randomised study) | ⨁◯◯◯ Very low^c,e^ |  |

***The risk in the intervention group** (and its 95% confidence interval) is based on the assumed risk in the comparison group and the **relative effect** of the intervention (and its 95% CI).

Explanations: a. wide 95% confidence intervals, b. Statistical heterogeneity (I^2^>30%), c. single study or small sample size, d. small number of events, e. lack of randomisation

Abbreviations: ADLs: activities of daily living, CG: control group, HABAM: hierarchical assessment of balance and mobility, IG: intervention group, MD: mean difference, SMD: standardised mean difference

# **Supplementary Table 4: Summary of Findings for Secondary Outcomes**

| Outcomes | **Anticipated absolute effects^*^** (95% CI) | | | Relative effect (95% CI) | № of participants (studies) | Certainty of the evidence (GRADE) |
| --- | --- | --- | --- | --- | --- | --- |
|  | **Risk with Usual Care** | | **Risk with Physical Activity** |  |  |  |
| **Falls** (Y/N)  During hospitalisation or within 3 months | 206 per 1,000 | **225 per 1,000** (77 to 502) | | **OR 1.12** (0.32 to 3.89) | 94 (3 non-randomised studies) | ⨁◯◯◯ Very low^d,e^ |
| **Readmission within 3 months** (Y/N) | 189 per 1,000 | **249 per 1,000** (32 to 766) | | **OR 1.42** (0.14 to 14.01) | 100 (2 non-randomised studies) | ⨁◯◯◯ Very low^b,d,e^ |
| **Length of Stay** assessed with days | LOS in control group ranged 7.85 to 18.00 | MD **1.10 lower** (3.14 lower to 0.93 higher) | | - | 110 (3 non-randomised studies) | ⨁◯◯◯ Very low^a,e^ |
| **Inpatient delirium** (Y/N) | 591 per 1,000 | **492 per 1.000**  (249 to 738) | | **OR 0.67**  (0.23 to 1.95) | 74  (2 non-randomised studies) | ⨁◯◯◯ Very low^a,c,e^ |

***The risk in the intervention group** (and its 95% confidence interval) is based on the assumed risk in the comparison group and the **relative effect** of the intervention (and its 95% CI).

Explanations: a. wide 95% confidence intervals, b. Statistical heterogeneity (I^2^>30%), c. single study or small sample size, d. small number of events, e. lack of randomisation

Abbreviations: MD: mean difference, OR: odds ratio

# **Supplementary Figure 1: Risk of bias by study**

**
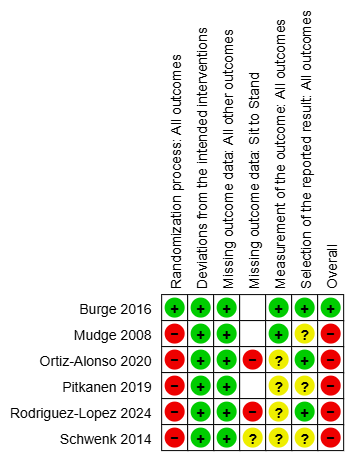
**

Footnote: The domain ‘Missing outcome data’ is split over columns 3 and 4, as the risk of bias judgement for the outcome measure ‘sit to stand’ differs to the other outcome measures (sit to stand is only available in three studies)

# **Supplementary Figure 2: Functional ability - independence in basic ADLs at discharge from hospital (change from pre-admission status)**


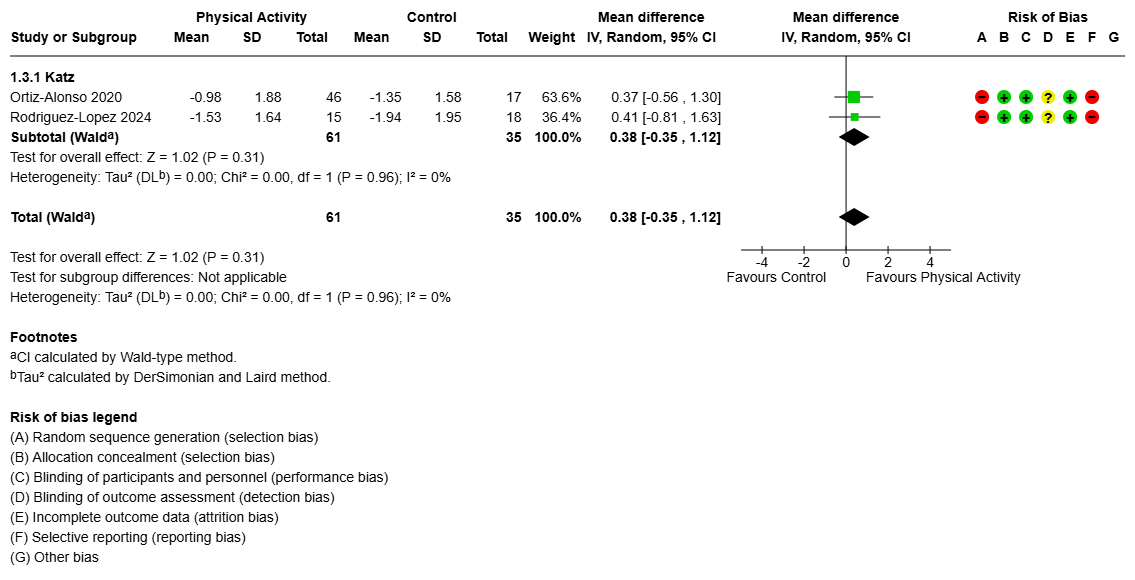


Risk of bias legend: (A) Randomisation process, (B) Deviations from intended interventions, (C) Missing outcome data, (D) Measurement of the outcome, (E) Selection of the reported result, (F) Overall bias

# **Supplementary Figure 3: Functional ability - independence in basic ADLs at three months post-discharge**


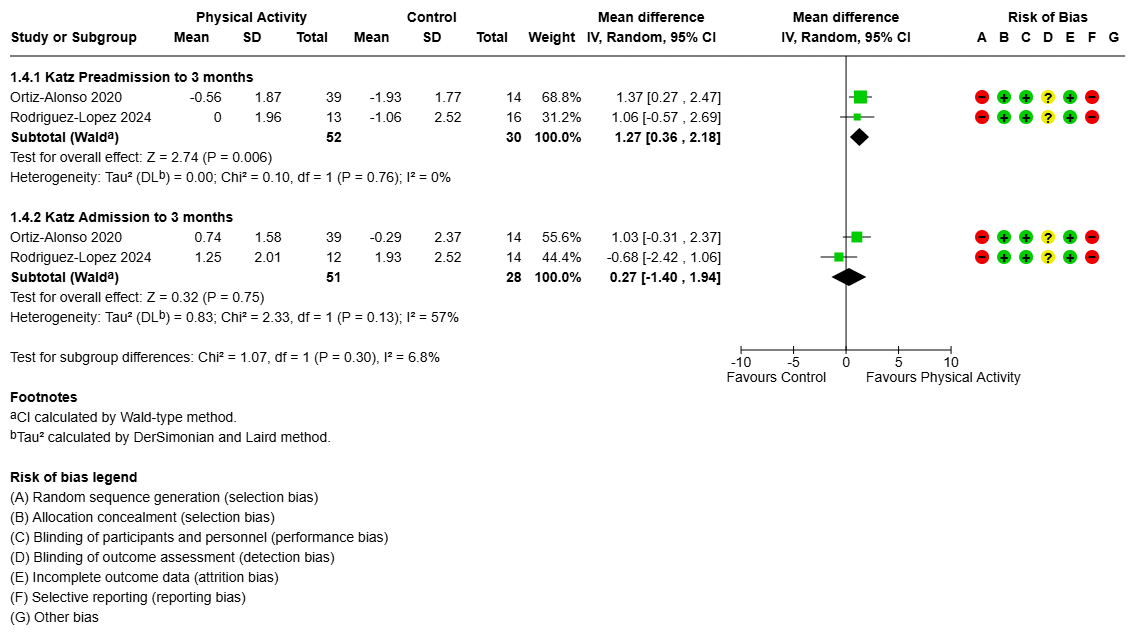


Risk of bias legend: (A) Randomisation process, (B) Deviations from intended interventions, (C) Missing outcome data, (D) Measurement of the outcome, (E) Selection of the reported result, (F) Overall bias

# **Supplementary Figure 4: Functional Ambulatory Classification at discharge**


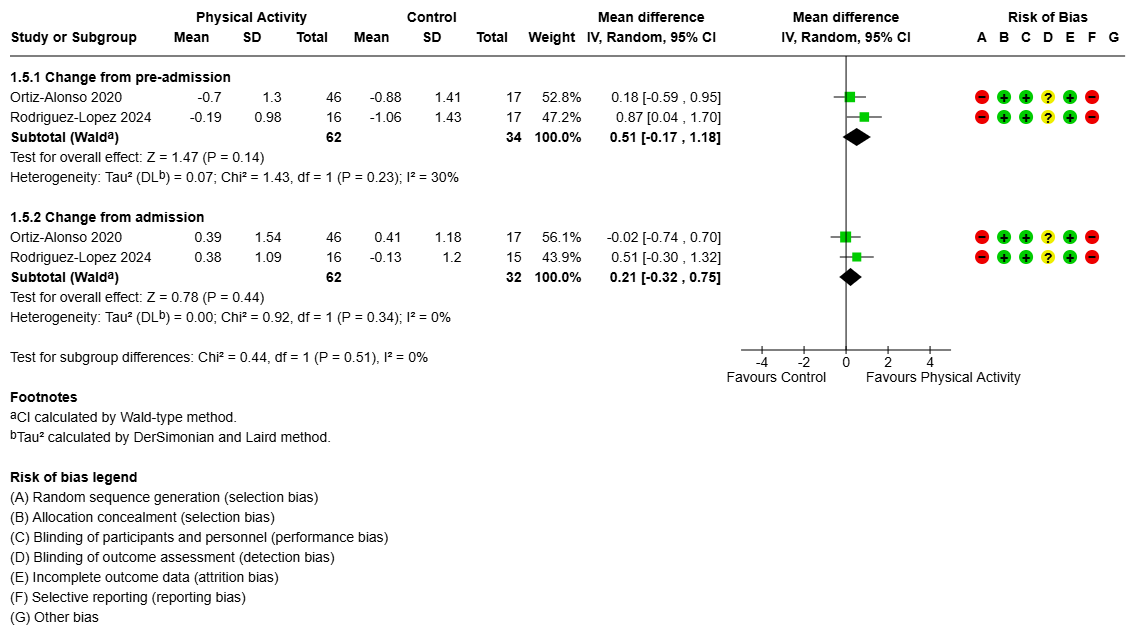


Risk of bias legend: (A) Randomisation process, (B) Deviations from intended interventions, (C) Missing outcome data, (D) Measurement of the outcome, (E) Selection of the reported result, (F) Overall bias

**Supplementary Figure 5: functional ability: Functional ambulatory classification (FAC) at three months post-discharge**

**
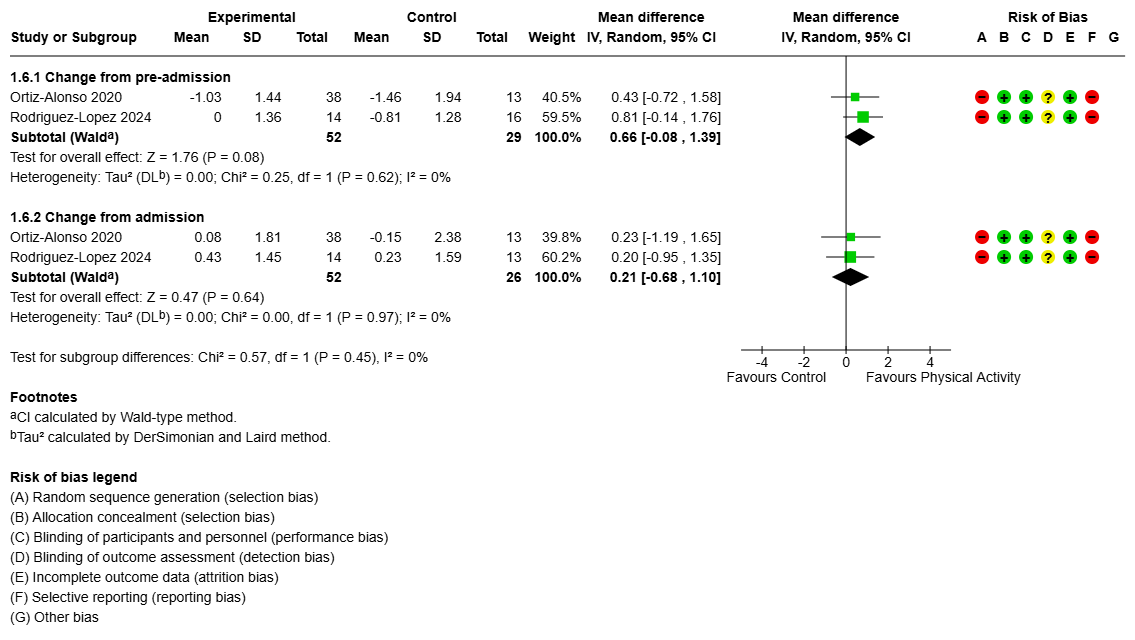
**

Risk of bias legend: (A) Randomisation process, (B) Deviations from intended interventions, (C) Missing outcome data, (D) Measurement of the outcome, (E) Selection of the reported result, (F) Overall bias

# **Supplementary Figure 6: Gait speed: distance (cm/sec) (change from baseline)**


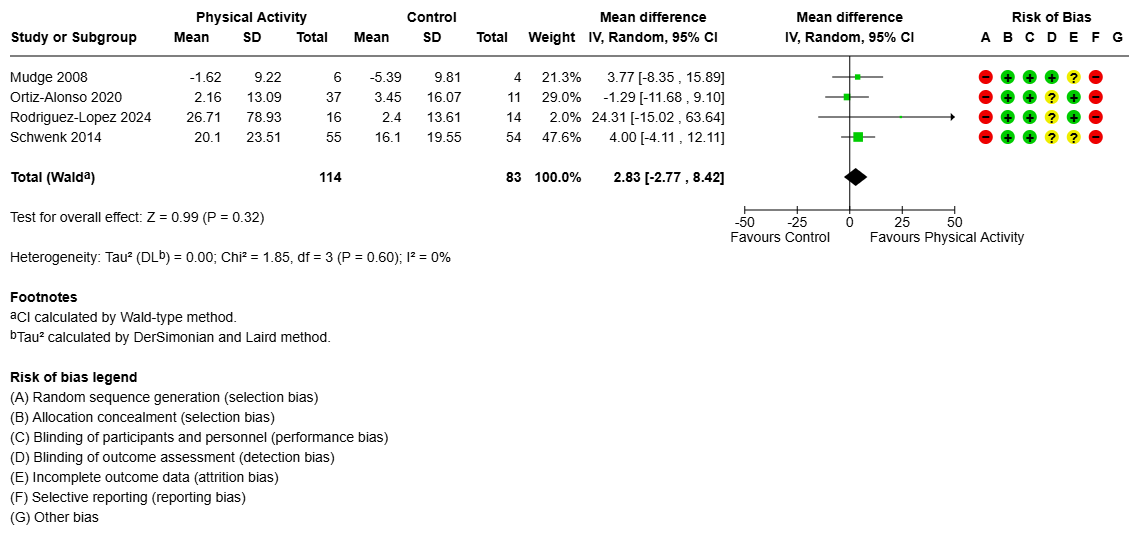


Risk of bias legend: (A) Randomisation process, (B) Deviations from intended interventions, (C) Missing outcome data, (D) Measurement of the outcome, (E) Selection of the reported result, (F) Overall bias

Different walking distances - all studies converted to cm/sec; **Mudge 2008:** timed up and go (rise from a chair and walk 3m and back), **Ortiz-Alonso 2020, Rodriguez-Lopez 2024**: 4 meters (SPPB test), **Schwenk 2014**: GAITRite-system: length 4.8 meters.

Standard deviation of change scores for Schwenk 2014 imputed.

**Supplementary Figure 7: Balance: SPPB subtest**


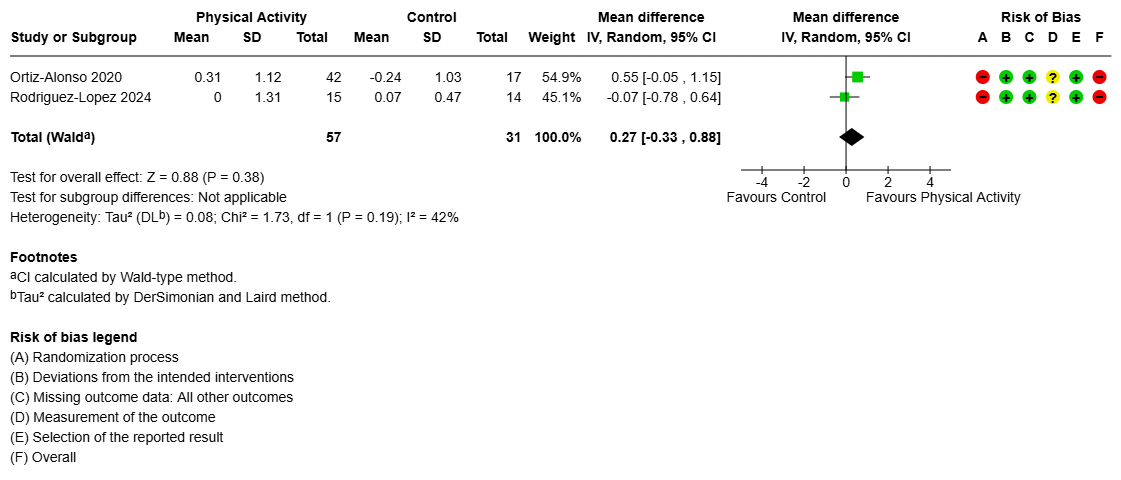


**Supplementary Figure 8: Number of fallers (either in hospital or within 3 months)**


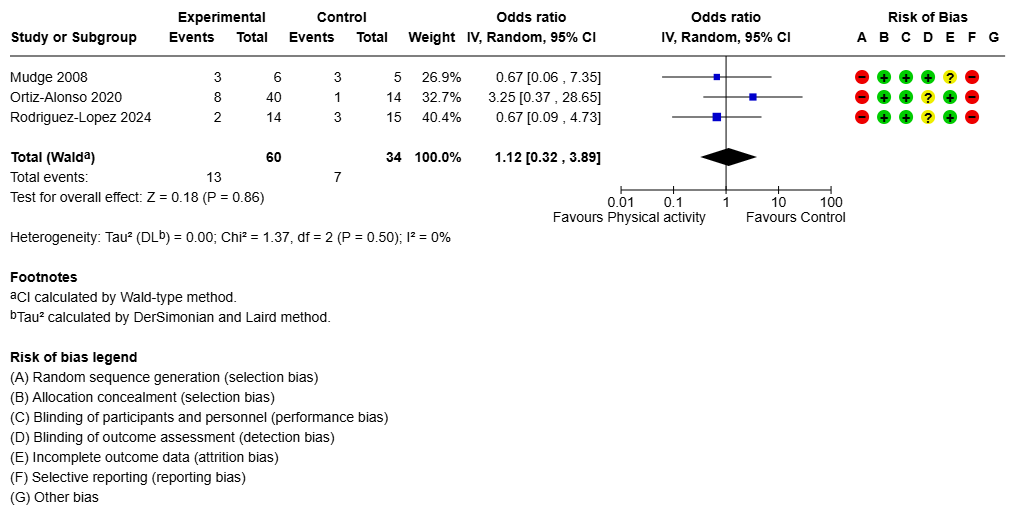


Risk of bias legend: (A) Randomisation process, (B) Deviations from intended interventions, (C) Missing outcome data, (D) Measurement of the outcome, (E) Selection of the reported result, (F) Overall bias

**Ortiz-Alonso 2020, Rodriguez-Lopez 2024**: Falls after discharge (within 3 months); **Mudge 2008**: Falls in hospital

# **Supplementary Figure 9: Readmission to hospital (within 3 months)**


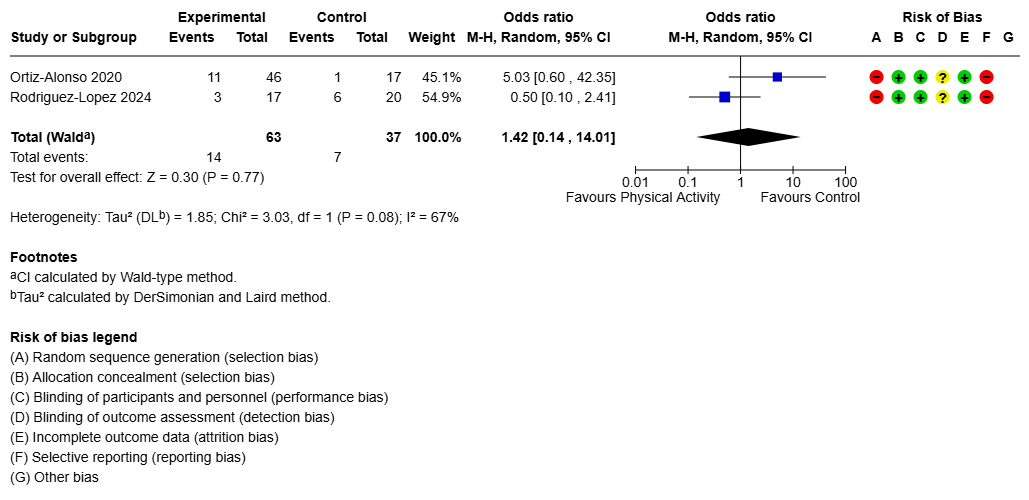


Risk of bias legend: (A) Randomisation process, (B) Deviations from intended interventions, (C) Missing outcome data, (D) Measurement of the outcome, (E) Selection of the reported result, (F) Overall bias

# **Supplementary Figure 10: Length of stay (days)**


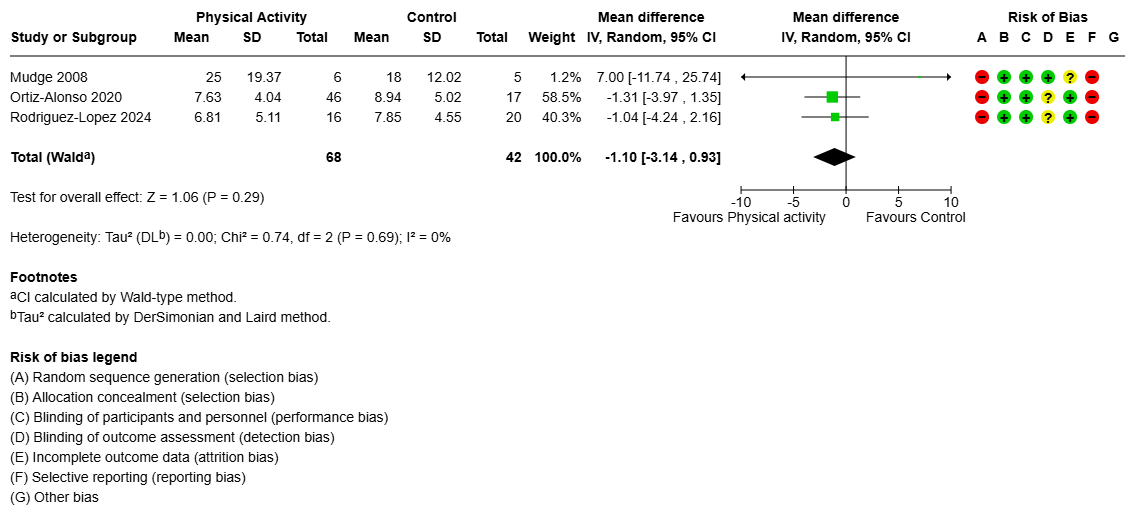
 Risk of bias legend: (A) Randomisation process, (B) Deviations from intended interventions, (C) Missing outcome data, (D) Measurement of the outcome, (E) Selection of the reported result, (F) Overall bias

# **Supplementary Figure 11: Incident delirium**


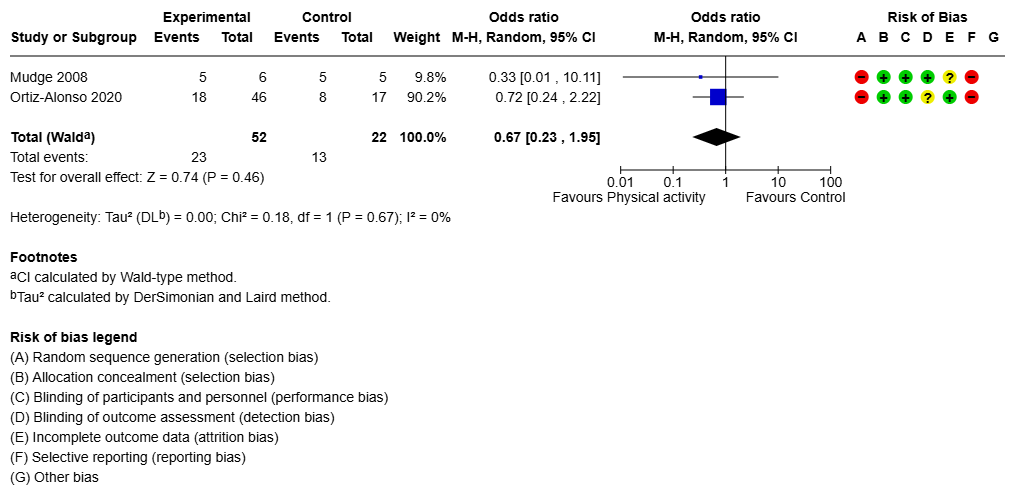
Risk of bias legend: (A) Randomisation process, (B) Deviations from intended interventions, (C) Missing outcome data, (D) Measurement of the outcome, (E) Selection of the reported result, (F) Overall bias
